# Decision Making in the Context of Paediatric Solid Organ Transplantation Medicine

**DOI:** 10.3389/ti.2022.10625

**Published:** 2022-07-14

**Authors:** Jenny Prüfe

**Affiliations:** Clinic for Pediatrics II, Essen University Hospital, Essen, Germany

**Keywords:** ethics, transplant ethics, psychology, solid organ transplant, paediatric

## Abstract

This manuscript aims to outline ethical, legal, and psychosocial key situations in the context of transplantation under special consideration of children. Besides being particularly vulnerable, children as minors by law are not meant to consent to whatever medical procedure is applied to them. Rather their next-of-kin and medical staff are to decide. In the context of transplantation thus it needs to be reflected under which circumstances a child can become an organ donor or receive an organ. This essay will not provide answers to current questions in transplantation medicine but provide an overview of present European practices and juxtapose divergent courses of action which are based on an assumed similar social-cultural background. Data are drawn from a systematic comparison of the various national organ transplantation laws and tissue acts. Ethical reflections are based on a thematically targeted literature search using PubMed Central and PhilPapers databases.

## The Child as an Organ Donor

In order to transplant an organ it needs an organ donor. Essentially, there are two options: living and deceased donor donation.

A living donor is a person who gives a kidney, or a part of liver, lung, or (in experimental cases) intestines to be donated to another person who is in need for such transplant. A healthy donor, informed consent, and a decision made voluntarily and without undue pressure are legal prerequisites for living donation and insisted on by the declaration of Helsinki [1]. To protect the potential donor, European transplantation laws or tissue acts require an independent assessment, e.g., by an ethics committee, to secure donor voluntariness and avoid organ trading. While some countries allow for non-directed, altruistic living donation, others ask for donor and recipient to be closely related either emotionally or by blood. With regards to the five principles of biomedical ethics, the principle of non-maleficence is challenged in the context of living donation: Each surgical procedure will not only cause pain but is a potentially life-threatening event to the donor. The medical risk varies considerably between organs: while a kidney-transplantation is considered a low risk procedure, the kidney does not regenerate resulting in a slightly elevated life-long increased risk to the kidney donor for developing arterial hypertension, proteinuria or even end stage renal failure [2]. In contrast, it is more dangerous to undergo liver donation, yet liver function will fully restore without long-term side-effects. Finally, liver transplantation is an ultimately live-saving procedure whereas dialysis offers a way to successfully bridge waiting time to transplantation. Yet, refusing a potential donor to donate may lead to emotional and psychological harm due to the stress which is immanent to living with a relative who is suffering from a chronic, life-limiting condition.

To date only five European countries allow for minors to act as living organ donors: In Belgium and the UK the individual’s maturity and capacity to make an autonomous decision and give fully informed consent is decisive. In Luxemburg, Norway and Sweden the parental permission is obligatory in addition to the assent of the potential donor [3].

Decision-making in the context of living-related donation poses a special challenge as the parent-child- and siblings-relationships are loaded with strong emotions, attachment, and perceived duties. As found by Russell et al. [4] merely raising the issue of live organ donation triggers an emotional cascade in parents which appears to be beyond their conscious control but leaves little room to freely decide. Rather, parents are restricted in their options given the thread to their child’s health and life, and their wish for optimal treatment. If now a minor is to donate to a sibling, the situation becomes even more complex as the moral obligation to protect the vulnerable needs to be recognised. The donor situation becomes more complicated as the potential donor might feel obliged to help the sibling in order to restore a normal family live. Simultaneously, parents may find themselves potentially sacrificing the health of one child for the other. This pressure arising from emotional involvement and perceived moral obligation may result in an implicit moral imperative which undermines true informed consent and freedom of choice. Apart from sufficient cognitive capacity to fully understand the situation, inner strength is needed to identify and possibly resist such potential coercion. The question re the child’s best interest appears unanswerable, weighing physical integrity against emotional burden.

A deceased donor in contrast is a person who donates any organ after his/her own death. The first challenge is to define death. In Europe the concept of brain death, as total and irreversible loss of all brain function, is widely accepted. However, by means of intensive care the brain-dead person will still have a functioning cardiovascular system. While most European countries demand full brain-death before proceeding into organ explantation, Poland, the UK, and Israel allow for brain-stem-death. The brain-stem controls basic regulatory functions such as breathing, blood pressure or heartbeat. The rational is that cessation of autonomous breathing is incompatible with life and thus brain-stem-death will result in full brain death as soon as artificial respiration is stopped.

But even the concept of brain-death as it was defined in the 1970 is disputed until today. For instance, in their “statement on brain death and the decision for organ donation” from 24th Febraury 2015 7 of 18 members of the German Ethics Council voted against declaring a brain-dead person as dead; rather they recommended to regard them as a dying person. By law this means, that they still have full personal rights, which are denied after death. While brain-death is a prerequisite to organ donation in most European countries and explicitly mentioned in the transplantation laws and tissue acts, the legal definition of death varies between countries or, in the case of Germany, is non-existent.

In addition to brain-death, some European Countries also accept donation after cardiac death (NHBD) for donation. In this case circulatory death is considered sufficient. While in the past NHB-donors were only acceptable for tissues (i.e., cornea, skin, bone, heart valves etc.), recent advances in medicine made it possible to recover kidneys, livers or lungs from humans following circulatory arrest.

The modified Maastricht Classification of donor after circulatory death defines 4 settings which vary regarding the circumstances of circulatory arrest and the potential use of organs [5]:

European legislation varies considerably regarding NHBD: Whereas in the UK in 2018 donation after cardiac arrest accounted for 40% of all deceased donation, it is strictly forbidden in Germany. While Italy quires a no-touch period of at least 20 min before organs can be recovered, most other countries accept 5 min as sufficient [6].

There are manifold reasons why NHBD transplantation is disputed. This includes the administration of drugs which do not benefit the donor, the risk to end resuscitation too early in order to retrieve organs, the active withdrawal of life-support, and potential harm to the dying person who might experience pain given that the brain is still functioning. In the case of Maastricht category II, it is necessary to perform cannulation and perfusion of a conserving liquid in order to preserve organs. This is done in high urgency, most likely before the donor’s next-of-kin can be asked for the assumed consent. Category III asks for an active withdrawal of treatment which will cause death. This can only be acceptable if the decisions regarding non-survivability of the health condition is correct and any further treatment will be futile. Decisions must be made in the best interest of the patient regardless the potential of organ retrieval. In any case, the definition of death based on the time of cardio-circulatory arrest appears arbitrary as the Institute of Medicine in 2000 [7] concluded: “existing empirical data cannot confirm or disprove a specific interval at which the cessation of cardiopulmonary function becomes irreversible.” Additionally, continuation of cardio-pulmonary-resuscitation can potentially restore cardiocirculatory activity even after hours, unless brain death has occurred. Finally, the need for high-end intensive care to preserve organs for donation may violate a person’s wish for an end-of-life-care without high-tech medicine, particularly if defined by a Do-Not-Resuscitate-order.

Independently of the type of deceased donor donation European legislations vary with regards to who is considered to be an organ donor. The crucial difference is the type of consent that is required. In the case of an opt-in system explicit consent is required. This means that the potential donor has declared his/her wish to donate during life-time. If the potential donor’s wish is not documented, the next-of-kin is asked for informed consent assuming the potential donor’s will. Nowadays most European countries operate on an opt-out system which is based on presumed consent. In this case anyone fulfilling the requirements for organ donation is considered a donor unless they have explicitly expressed their unwillingness to donate during life-time (dissent solution). The biggest challenge to the latter is a potential to undermine autonomy and to force a decision. While proponents of this approach claim that anyone is free of choice to opt out, opponents argue that the decision to (not) donate is not a dichotomous choice. Rather, there is a need for a third option which leaves room for the potential donor to delegate the decision to family members or a trusted person.

In 2021, Eurotransplant accounted for 55 deceased donors younger than 16 years which represents 2.9% of all deceased donors in the Eurotransplant countries [8]. The UK, which allows for brain-stem-death as valid criterion in adults excludes children younger 2 months of age from donation as it is considered rarely possible or even impossible to confidently diagnose death as a result of cessation of brain-stem reflexes in this age [9]. While infant donation thus is not possible in the UK, the import of infant organs from other countries is legally and socially accepted.

In countries with an opt-in system paediatric donation is different from adult donation to the extent that the parent or legal guardian always has to authorise the donation, irrespective the deceased minor’s opinion. However, the minimum age required to declare one’s intention varies considerably and can be as low as 12 years (NHS Scotland). In countries with an opt-out system, there is uncertainty how this can be applied to children without incapacitating the parents. In most cases the regulations are thus suspended and do not apply to minors and adults who lack the capacity to understand the implications; again, the legal guardian’s consent is required.

Although parental consent is legally requested in case of paediatric donation, it needs to be questioned how informed such consent can be under the given circumstances. Little is known about how organ donation might conflict with parental expectations. Particularly, if a child’s death does not occur suddenly in the context of an accident but comes gradually due to a progressive life-limiting condition parents frequently wish for the child to stay at home or to be hold when death occurs. This conflicts with the need for high-end intensive care necessary for organ recovery. Finally, one needs to ask whether merely raising the question of donation may cause further harm to the bereaved ones if not placed appropriately. This might be particularly the case, when parents find themselves in the stress-field of weighing the own and their child’s assumed needs against the societal needs and perceived moral obligations.

Data show that parents of a minor decide differently than relatives of potential adult donors: In 2018 the NHS Blood and Transplant reports a consent in 48% of the cases of minor donors as compared to the average consent rate of 66% across all ages.

In any case, parents are approached in the moment of utmost tragedy and possibly largest emotional defencelessness in order to make an undirected gift to help some unknown other. Bennett et al. [10] report that clinicians fail to refer patients to the relevant donation organisation in 23% of all withdrawal-of-therapy cases. Numbers were found to be lowest in children age 1 month and younger with a non-referral rate of 39%. While Hawkins et al. [11] identify medical reasons such as perceived medical unsuitability, it is also reported that medical staff feels unsure about if and how to approach the relevant families [12]. It is disputable whether such structural barriers to donation are acceptable, given that they do not only deny a family the chance to donate but also might deny organs to patients on the waiting list.

## The Child as an Organ Recipient

Since the first solid organ transplantations to children in the 1960s [13–15] paediatric transplantation medicine has come a long way. The transplantation of kidney, liver, heart, and lung has become a routine procedure to save and prolong lives of children with terminal organ failure even in infancy.

Legally, there are no clear restrictions as to under which circumstance a child may or may not receive a vital organ. Technically, there are some constraints based on the anatomic conditions. Questions however arise frequently in terms of.

- the child’s ability and necessity to at least assent to transplantation and the related therapeutic procedures,
- the justifiability of organ transplantation in children with severe mental disabilities or crippling conditions where transplantation may result in extended suffering,
- the necessity of a good enough social and/or familiar support.

The need to assent becomes relevant with age. While most policies require paediatric patients to come of age in order to express their free will to most medical procedures, organ transplantation is different to the extent that it asks maximum commitment of the transplant recipient. If a young person mentally rejects the organ, non-adherent behaviour and subsequent biological rejection of the organ become more likely. Forcing a child into transplantation without the ability to secure consequent maintenance treatment means to potentially withhold an organ from someone who might have been more ready to accept it.

However: when is a child old enough to encompass the consequences of transplantation or its refusal and what happens if a child’s wish conflicts with a child’s wellbeing? Claiming that a child’s decision may not be in the child’s best interest asks for who is to define the best interest. Not only that “best interest” is a vague construct, it is susceptible to the bias and prejudice of the person interpreting this construct.

One of the most prominent cases on child decision-making in recent history is the one of Hannah Jones who at the age of 13 years denied heart transplantation. Hannah had suffered leukaemia at 4 years of age and subsequently developed severe cardiomyopathy as a complication to chemotherapy. As a teenager she decided that she no longer had the strength to fight her health conditions and rather wanted to spend her limited life-time outside hospitals and aggressive treatment. While her parents accepted her wish the medical team did not. Consequently, the case was meant to be taken to High Court aiming to define best interest and the acceptability of Hannah’s wish. Legal actions were however dropped, after a member of the local child protection team advised the primary care trust that Hanna was competent to make her decision.

The United Nations Convention on the Rights of the Child clearly state in article 12 that:

“States Parties shall assure to the child who is capable of forming his or her own views the right to express those views freely in all matters affecting the child, the views of the child being given due weight in accordance with the age and maturity of the child.”

Thus the question is not whether a child is old enough but capable of forming an own view and understanding the consequences. Above all this requires a constant dialogue between health care providers, patient, and family in order to hear the wishes, understand the needs, answer questions, and judge on the relevant capacities.

The justifiability of transplantation in multiply disabled children is highly debated and there is no consent across European approaches. While general disability-based discrimination is forbidden across Europe, reasons to deny access to transplantation include a potentially reduced life-expectancy, a lack of improvement in terms of quality of life, or a lacking ability to comply with the complex treatment following transplantation [16, 17].

Research shows that allograft function and survival of children with severe developmental delay but no other conflicting health condition does not significantly differ from the outcome of other recipients. If at all, adherence appears to be better and immunosuppressant trough-levels more stable in paediatric organ recipients with developmental delay. This is attributed to the continuous and consequent care provided by the parents or relevant custodian as well as to the lack of pubertal opposition. Thus, a possible decision against donation in case of mental disability is not based on possible medical outcome but on assumed concerns with regards to psycho-social management [16, 18].

Clinical practice differs in case of comorbidities in addition to developmental difficulties, particularly when the comorbidity causes uncontrollable suffering or significantly shortens life-expectancy [19]. Overall transplant-results, policies, and medical approaches vary considerably. In this light, individualised assessments which respect the patients’ and relatives’ wishes, and include an external review of other experts in the field become indispensable. The aim needs to be to balance the benefits and burdens on a case to case base [20].

Social support is essential in paediatric organ donation. Allograft maintenance asks for frequent visits to specialist doctors, home assessments of bodily functions, and a strict, life-long daily medication regime. Additionally, transplantation may interfere with developmental experiences and alienate a child from relevant peers both in appearance as well as in behaviour and psychosocial development. In cases where social support is lacking and follow-up care cannot be secured the success of transplantation is at significant risk. Facing the overall lack of available donor organs it is disputable, whether an organ can be provided to a patient with a poor outlook. Concurrent obligations occur with the potential recipient on the one side, and other candidates on the waiting list on the other side.

As in the case of developmental delay or comorbidities, lacking social support is not a strict exclusion-criterion to transplantation but demands careful consideration. Given the complexity of the situation Dionne et al. [21] recommend accounting for contextual and societal factors when considering organ donation. While it is comprehensible to provide a scarce good such as an organ only under the provision of a good perspective, social support requirements may reinforce social injustice further disadvantaging children from complex social context. Thus one might argue that the provision of sufficient social support in such cases needs to be improved instead of excluding the already marginalised.

In summary organ donation and transplantation are no straight forward processes by the means of psychology, sociology or ethics. Some challenges may only be approachable on an individual base and ask for thorough frameworks that facilitate just decision making. Other challenges may be addressed by legal guidelines however - as indicated - jurisdiction can vary considerably even in what is thought a common European socio-cultural background. Broadening the discussion to other geographical, cultural, religious or societal contexts might add to the complexity of the topic by adding further ethical ideas and legal frameworks, e.g., the acceptance of organ trading, and drawing a widely heterogeneous picture.

Advances in medicine have the potential to raise chances but with increasing options also more challenges may occur. An interdisciplinary discourse is needed to tackle the issues addressed.

## Data Availability

The original contributions presented in the study are included in the article/supplementary material, further inquiries can be directed to the corresponding author.
